# Draft Genome Sequence of Pseudomonas sp. Strain MWU15-20650, Isolated from Wild Cranberry Fruit in the Cape Cod National Seashore

**DOI:** 10.1128/mra.00547-22

**Published:** 2022-07-18

**Authors:** Tyler Sholl, Scott Soby

**Affiliations:** a Biomedical Sciences, College of Graduate Studies, Midwestern University, Glendale, Arizona, USA; b College of Veterinary Medicine, Midwestern University, Glendale, Arizona, USA; University of Arizona

## Abstract

Pseudomonas sp. strain MWU15-20650 was isolated from wild cranberry fruit surfaces in the Cape Cod National Seashore. The draft genome is 6.2 Mbp, with a G+C content of 59%, and contains predicted genes for type VI secretion systems and an *N*-acyl-homoserine lactone acylase. The closest known relative is Pseudomonas haemolytica.

## ANNOUNCEMENT

The wild cranberry bogs of the Cape Cod National Seashore represent an understudied ecosystem. Because little to no fungal disease is apparent in wild bogs, the bacteria associated with healthy wild cranberry flowers and fruits may represent a resource for natural disease suppression that can be translated to cultivated plants (1). A dominant group of these bacteria is Pseudomonas spp., some of which can produce secondary metabolites with biological activity against pests and pathogens (2–4). Here, we present the draft genome sequence of Pseudomonas sp. strain MWU15-20650, which was isolated as part of a survey of berry and flower surfaces in July 2015 during late flowering to early fruit in wild cranberry bogs in the Cape Cod National Seashore in Provincetown, Massachusetts (42.062065N, 70.118679W). Cranberry fruits were vortexed in sterile water, and the rinsate was plated on King’s medium B (KMB) agar containing 50 μg mL^−1^ each of ampicillin and cycloheximide. Fluorescent colonies were streaked for isolation on fresh KMB agar, single colony purified three times, and stored at −80°C in 34% glycerol. MWU15-20650 was then grown in KMB broth overnight for genomic DNA (gDNA) isolation (DNeasy blood and tissue kit; Qiagen). Libraries were generated with a HyperPlus library preparation kit (KK8514; Kapa Biosystems, Roche, USA). DNA was enzymatically fragmented to ~500 bp, end repaired, and A-tailed as described in the Kapa Biosystems protocol. Illumina-compatible adapters with unique indexes (00989130v2; Integrated DNA Technologies) were individually ligated to each sample, the samples were cleaned with KAPA pure beads (KK8002; Kapa Biosystems) and amplified with HiFi enzyme (KK2502; Kapa Biosystems). Fragment sizes were determined on an Agilent TapeStation system and quantified using quantitative PCR (qPCR) (KAPA library quantification kit [KK4835]) on a QuantStudio 5 system (ThermoFisher Scientific). The library was multiplex pooled for sequencing in a 2 × 250-bp flow cell on the Illumina MiSeq platform. Raw reads were assembled and quality controlled in the PATRIC (http://patricbrc.org) Comprehensive Genome Analysis pipeline v3.6.12 (5) using default settings except for the automated trimming function (which was set to true), Unicycler v0.4.8 (6), and two rounds of polishing with Pilon v1.23 (7). The pipeline includes quality control with Trim Galore v0.4.0 (https://www.bioinformatics.babraham.ac.uk/projects/trim_galore) (8) and annotation with RASTtk (9). Strain MWU15-20650 had a genome size of 6,082,616 bp assembled into 86 contigs from 1,094,993 reads, with a total read length of 530,079,409 bp. The G+C content was 59.92%, and the *N*_50_ value was 122,598 bp, with 87× coverage. The isolate was placed in the genus Pseudomonas by genome BLAST distance phylogeny (GBDP) using the TYGS online tool (https://tygs.dsmz.de), but it is not a member of any known species, being most closely related to Pseudomonas haemolytica (digital DNA-DNA hybridization [dDDH_d4_], 35.4%). The genome contains predicted genes for type VI secretion systems and an *N*-acyl-homoserine lactone acylase, indicating that MWU15-20650 may affect members of the microbiome directly by competition (10) and indirectly by interference with their quorum-sensing systems (11).

### Data availability.

This whole-genome shotgun project has been deposited in DDBJ/EMBL/GenBank under the accession number JALJFG000000000 for Pseudomonas MWU15-20650. The version described in this paper is version JALJFG000000000.1, with BioProject accession number PRJNA691338 and BioSample accession number SAMN26725498. The Sequence Read Archive (SRA) accession number is SRR18508442. RASTtk annotations are available under open license at Zenodo (https://zenodo.org/record/6392140#.YpFJpqDMKUk).
